# Clinical Impact of the Early Use of Monoclonal Antibody LY-CoV555 (Bamlanivimab) on Mortality and Hospitalization Among Elderly Nursing Home Patients: A Multicenter Retrospective Study

**DOI:** 10.7759/cureus.14933

**Published:** 2021-05-10

**Authors:** Mohammud M Alam, Saborny Mahmud, Sandeep Aggarwal, Sawsan Fathma, Naim Al Mahi, Mohammed S Shibli, Siddiqi M Haque, Sharothy Mahmud, Ziauddin Ahmed

**Affiliations:** 1 Medicine, Donald and Barbara Zucker School of Medicine at Hofstra/Northwell, Hempstead, USA; 2 Medicine, Icahn School of Medicine at Mount Sinai, New York, USA; 3 Nephrology, University of Pennsylvania, Philadelphia, USA; 4 Internal Medicine, Swedish Hospital, Chicago, USA; 5 Genomics Research Center, AbbVie, Chicago, USA; 6 Internal Medicine, Glen Cove Center for Nursing and Rehabilitation, Glen Cove, USA; 7 Biological Sciences, Barnard College at Columbia University, New York, USA; 8 Medicine, Temple University, Philadelphia, USA

**Keywords:** covid-19, covid-19 treatment, sars-cov-2, ly-cov555, bamlanivimab, monoclonal antibody, long-term care facility, nursing home, non-hospital setting

## Abstract

Importance

Coronavirus disease 2019 (COVID-19) outbreaks are frequent occurrences in nursing homes and long-term care facilities (LTCFs), resulting in subsequent hospitalization and death.

Rationale

Virus-neutralizing monoclonal antibodies demonstrate a significant decrease in both viral load and hospital transfer rate among patients with mild-to-moderate COVID-19 infection.

Objective

To assess the clinical outcomes of COVID-19 patients with mild-to-moderate symptoms in LTCFs who received LY-CoV555 as compared to those who did not receive this treatment.

Design

Retrospective case-control study and logistic regression analysis.

Setting

LTCFs in New York.

Participants

Two-hundred forty-six (246) LTCF patients diagnosed with mild-to-moderate COVID-19 infection with positive COVID-19 polymerase chain reaction (PCR) from November 15, 2020, to January 31, 2021.

Methods

Two-hundred forty-six (246) COVID-19 patients were identified from electronic medical records, out of which 160 cases were exposed to LY-CoV555 treatment (700 mg single dose, intravenous infusion). Eighty-six (86) patients were unexposed controls who did not receive monoclonal antibodies, LY-CoV555.

Outcome

We assessed the odds of death and hospitalization of exposed cases as compared to unexposed controls. Using logistic regression analysis, we also assessed the risk factors associated with these outcomes in the entire sample population.

Results

The mean age of the entire sample was 82.4 years. Fifty-two percent (52%) of patients (n = 129) were female and 48% (n = 117) were male. The mean ages of the exposed group and the unexposed group were 81 years and 84 years, respectively. At the end of the study, 92% (148/160) of the exposed group were alive or not transferred to the hospital as compared to 79% (68/86) patients of the unexposed group (OR 3.23, 95% CI: (1.48, 7.31), p-value = 0.0032). Three percent (3%; 5/160) of patients died in the exposed group compared to 10% (9/86) of patients who died in the unexposed group (OR = 0.25, 95% CI: (0.1, 0.85), p-value = 0.0257). Four point thirty-seven percent (4.37%; 7/160) of patients in the exposed group and 10.46% (9/86) of patients in the unexposed group were transferred to the hospital (OR = 0.35, 95% CI: (0.15, 1.08), p-value = 0.0793).

Conclusion

Early treatment with monoclonal antibody LY-CoV555 is associated with decreased mortality among high-risk patients with mild-to-moderate COVID-19 infection in LTCFs. Although not statistically significant, there was a trend towards a lower risk of hospitalization in patients treated with LY-CoV555.

## Introduction

The coronavirus disease 2019 (COVID-19) pandemic has spread rapidly, disrupting the lives of millions of people in the world and placing an overwhelming burden on the U.S healthcare system [1]. As of March 31, 2021, there have been over 30 million COVID-19 cases and over 551,000 deaths in the U.S. [2], and nursing home residents and staff account for around 40% of COVID-19-related deaths [3]. Due to their congregate setting and multiple medical conditions, residents in long-term care facilities (LTCFs) are at high risk for the progression of severe COVID-19 infection, hospitalization, and death. Multiple treatment regimens have been implemented to treat hospitalized patients with COVID-19, including antimalarial drugs [4], antiviral agents [5], anthelmintics [6], immunomodulators [7], glucocorticoids [8], and convalescent plasma [9]. However, treatment options for out-patients with COVID-19 infection are limited. Our group initially used doxycycline and hydroxychloroquine to treat COVID-19 infection in LTCF residents [10] and later used doxycycline alone [11]. 

The COVID-19 virus enters cells through binding of its spike protein to angiotensin-converting enzyme 2 (ACE2) receptors on target cells [12]. Recently, several studies in animal models with virus-neutralizing monoclonal antibodies for COVID-19 infections have shown promising results [13]. The LY-CoV555 monoclonal antibody binds to the receptor-binding domain of the viral spike protein. Studies demonstrate that utilizing monoclonal antibodies for mild-to-moderate symptoms of COVID-19 infections in outpatient settings reduces the viral load, improves symptoms, and prevents hospitalization [14-16]. Here, we report the clinical findings of high-risk patients in LTCFs with mild-to-moderate COVID-19 who received LY-CoV555 treatment.

## Materials and methods

Study design

We conducted a retrospective chart review of 246 LTCF residents, diagnosed with mild-to-moderate COVID-19 infection with a positive COVID-19 polymerase chain reaction (PCR) test result between November 15, 2020, and January 31, 2021. Out of the 246, 160 patients were treated with the virus-neutralizing monoclonal antibody LY-CoV555 (Bamlanivimab), a 700 mg single dose infusion over one hour within 48 hours after the initial diagnosis. Patients in the exposed group or patients’ families were fully informed about the risks and benefits of LY-CoV555 and provided informed consent before starting this treatment. Safety was assessed in all patients. Eighty-six patients did not receive LY-CoV555 treatment (patients were not prescribed it by their primary care physicians, patients or their families did not consent, or for other reasons). Oversight medical boards and corporate clinical services approved this study.

Statistical analysis

The data were collected from institutional electronic medical records and saved in a secure portable computer. Data were tabulated as the mean and standard deviation (SD) for continuous variables (e.g., blood urea nitrogen (BUN), C-reactive protein (CRP), D-dimer, etc.) and percentages and numbers for categorical variables (e.g., sex, race, obesity, etc.). Comparisons between patients exposed and not exposed to monoclonal antibodies were conducted using a two-sample t-test for continuous variables and Fisher’s exact test for categorical variables. All tests were two-tailed with a statistical significance level of α = 0.05. Binary logistic regression analysis was conducted to assess the association between risk factors and mortality. We selected our model through stepwise logistic regression and assessed its goodness-of-fit using Akaike Information Criterion (AIC). The R programming language (version 4.0.2; R Foundation for Statistical Computing, Vienna, Austria) was used for all statistical analyses.

## Results

The mean age of all 246 patients was 82.4 years, ranging from 58 to 100 years. Fifty-two percent (52%) (n=129) of patients were female and 48% (n=117) were male. The mean ages of the exposed and unexposed groups were 81 years and 84 years, respectively. Approximately 5% (n=14) total patients died and approximately 6% (n=16) patients were transferred to the hospital due to clinical deterioration.

Ninety-two percent (92%; 148/160) patients of the exposed group were alive or not transferred to hospital as compared to 79% (68/86) of the unexposed patients (OR 3.23, 95% CI: (1.48, 7.31), p-value = 0.0032). Three percent (3%; 5/160) patients died in the exposed group as compared to 10% (9/86) patients in the unexposed group (OR = 0.25, 95% CI: (0.1, 0.85), p-value = 0.0257), indicating that patients exposed to LY-CoV555 are statistically significantly less likely to die than the patients who were not exposed to the monoclonal antibody. Exposed patients seem to have lower odds of hospitalization although this finding is not statistically significant at the 5% level.

Furthermore, the odds of having altered mental status (AMS) is significantly lower in the exposed patient group, whereas higher odds of coronary artery disease (CAD) were observed among patients in the exposed group. Variables such as obesity, congestive heart failure (CHF), chronic obstructive pulmonary disease (COPD), diabetes, and side effects were not associated with exposure status (Table 1).

**Table 1:** Categorical characteristics, clinical outcomes, and clinical features of patients who were exposed and patients who were not exposed to LY-CoV555 treatment, along with their respective odds ratios and p-values

Categorical Variables		Exposed Patients (n=160)	Unexposed Patients (n=86)	Odds Ratio (95% Confidence Interval)	P-value
Death	No	96.9% (n=155)	89.5% (n=77)	0.25 (0.1, 0.85)	0.0257
Hospital transfer	No	95.6% (n=153)	89.5% (n=77)	0.35 (0.15, 1.08)	0.0793
Sex	Female	48.8% (n=78)	59.3% (n=51)	1.47 (0.9, 2.58)	0.1168
	Male	51.2% (n=82)	40.7% (n=35)		
Race	Black	16.9% (n=27)	18.6% (n=16)	0.93 (0.37, 3.18)	0.8773
	Hispanic	8.1% (n=13)	8.1% (n=7)		
	White	75% (n=120)	73.3% (n=63)		
Altered mental status (AMS)	No	68.1% (n=109)	25.6% (n=22)	0.16 (0.09, 0.29)	<0.0001
Obesity	No	55.6% (n=89)	48.8% (n=42)	0.73 (0.45, 1.27)	0.2894
Ventilator	No	95.6% (n=153)	94.2% (n=81)	0.61 (0.23, 2.25)	0.6210
Chest pain	No	76.9% (n=123)	77.9% (n=67)	1 (0.56, 1.96)	0.8624
Coronary artery disease (CAD)	No	41.9% (n=67)	57% (n=49)	1.76 (1.08, 3.1)	0.0248
Congestive heart failure (CHF)	No	72.5% (n=116)	62.8% (n=54)	0.62 (0.37, 1.12)	0.1213
Chronic obstructive pulmonary disease (COPD)	No	60% (n=96)	66.3% (n=57)	1.25 (0.76, 2.25)	0.3375
Cough	No	11.9% (n=19)	18.6% (n=16)	1.59 (0.83, 3.47)	0.1600
Malaise/Weakness	No	22.5% (n=36)	22.1% (n=19)	0.95 (0.54, 1.87)	0.9864
Diarrhea	No	79.4% (n=127)	81.4% (n=70)	1.06 (0.58, 2.17)	0.7157
Diabetes	No	56.2% (n=90)	59.3% (n=51)	1.09 (0.67, 1.92)	0.6487
Side effect	No	95.6% (n=153)	90.7% (n=78)	0.44 (0.18, 1.46)	0.2364

Comparing patients who were exposed to LY-CoV555 to patients who were not exposed, after treatment, statistically significant mean differences were observed in the resolution of fever, resolution of shortness of breath (SOB), high lactate dehydrogenase (LDH), age, and pulse oximetry (POX). High LDH along with time to resolution of both fever and SOB tended to be lower on average in the exposed group, whereas the mean POX after treatment is higher in the exposed group. Other variables, such as high D-Dimer, high creatinine, and high troponin, were not significantly different between exposed and unexposed groups (Table 2).

**Table 2:** Clinical features with continuous measurements for patients who were exposed to and patients who were not exposed to LY-CoV555 treatment, along with their respective mean values and p-values

Continuous Variables	Mean in the Exposed Group	Mean in the Unexposed Group	P-value
Time to resolution of fever (Days)	1.98 (0.07)	3.9 (0.13)	<0.0001
Time to resolution of shortness of breath (Days)	2.58 (0.07)	3.91 (0.13)	<0.0001
High lactate dehydrogenase (LDH)	578.33 (17.02)	693.09 (29.97)	0.0011
Pulse oximetry (POX) after Rx	95.51 (0.27)	92.98 (0.76)	0.0023
Age	81.42 (0.68)	84.42 (0.89)	0.0084
High D-dimer	3.41 (0.3)	4.1 (0.43)	0.1933
High creatinine	1.65 (0.04)	1.75 (0.06)	0.2068
High troponin	0.62 (0.11)	0.73 (0.19)	0.5861
High ferritin	516.26 (19.57)	503.5 (25.11)	0.6891
High regular C-reactive protein (CRP)	9.22 (0.61)	9.05 (0.85)	0.8693
Pulse oximetry (POX) before Rx	95.12 (0.17)	95.09 (0.26)	0.9338
High blood urea nitrogen (BUN)	35.68 (1.01)	35.74 (1.44)	0.9729

We used binary logistic regression analysis to assess the association between risk factors and mortality. High D-dimer (OR = 1.201, 95% CI: (1.094, 1.319), p-value = 0.0001), delay in resolution of SOB (OR = 1.914, 95% CI: (1.239, 2.956), p-value = 0.0034), chest pain (OR = 3.709, 95% CI: (1.246, 11.041), p-value = 0.0185), diabetes (OR = 3.587, 95% CI: (1.094, 11.748), p-value = 0.0348), and high troponin (OR = 1.224, 95% CI: (1.007, 1.488), p-value = 0.0424) were associated with increased mortality. A higher POX after treatment (OR = 0.783, 95% CI: (0.716, 0.855), p-value = <0.0001) tends to reduce mortality. Other variables, such as BUN, creatinine, CRP, ferritin, and LDH were not associated with mortality.

Four point three-seven (4.37%; 7/160) patients who received monoclonal antibodies had infusion-related side effects. These side effects were mild and included generalized weakness, nausea, chills, and diarrhea, and one patient had a mild rash. Other adverse events were self-limiting, and no interventions or hospitalizations were required. Neither anaphylaxis nor serum sickness was noted in the exposed group.

## Discussion

COVID-19 remains a public health emergency. Despite ongoing efforts pertaining to reducing community spread by infection control barriers, social distancing measures, and widespread international vaccination efforts, the treatment of COVID-19 patients with acute illness remains a challenge. While direct cytopathic effects of the COVID-19 virus continue to be matters of investigation, there seems to be a consensus regarding the activation of endogenous immunological [17], inflammatory [18-19], and pro-coagulation pathways [20-21], which lead to adult respiratory distress syndrome (ARDS) and multiorgan failure as a large component of morbidity and mortality in severe COVID-19 syndrome. Thus, managing severe COVID-19 disease becomes complicated as treatment mandates the use of immunosuppressive agents; virus neutralization is not very effective at this stage.

A more reasonable approach is to prevent severe COVID-19 disease in outpatient management, either by active immunization through vaccination [22-23] or by viral neutralization in mild-to-moderate COVID-19 disease, especially in high-risk individuals. Wider availability of early treatment interventions and improving outpatient management of COVID-19 syndrome is critical in order to alleviate the overburdened healthcare system.

Passive transfer of virus-neutralizing antibody, using convalescent plasma, has been attempted in hospitalized patients who are at high risk of progression to severe COVID-19 syndrome [24-25]. A retrospective study showed improved survival in patients with high COVID-19 antibody levels compared with patients with low levels [26]. Although reassuring, a big logistical problem remains to obtain sufficient amounts of antibody titer plasma donors.

Monoclonal antibodies target the receptor-binding domain on the COVID-19 virus’ spike protein that binds to the ACE2 receptor [27], a receptor found on numerous cell types. Viral infection is mediated by the interaction between the viral spike and the ACE2 receptors found on numerous cell types and neutralizing monoclonal antibodies block this event [28]. Two independent groups of investigators have reported the findings of virus-neutralizing monoclonal antibodies. Studies assessing Bamlanivimab (LY-CoV555) and Casirivimab and Imdevimab together (REGN-COV2) - published in 2020 in October and December, respectively - showed a lowering of viral load and reduced rate of hospitalization when given to the patients with confirmed COVID-19 tests [15-16]. These results have led to a US-Food and Drug Administration (FDA) emergency utilization approval of virus-neutralizing antibodies for the treatment of mild-to-moderate COVID-19 infection in adults and pediatric outpatients >12 years of age. These therapies are additional tools that can be used to fight COVID-19 infection to prevent hospitalization, severe COVID-19 syndrome, and death.

Our study focuses on a population of elderly LTCF residents who, due to their co-morbidities and proximity to other patients, remain at a higher risk of poor COVID-19 outcomes. We conducted a retrospective analysis from the electronic medical records data of 246 patients in multiple LTCFs in New York. We compared baseline factors and outcome differences in the patients who received the COVID-19 neutralizing antibody, LY-CoV555, to those who did not receive it. LY-CoV555 treatment for patients at high risk for disease progression in LTCFs is associated with lower odds of mortality as compared to patients who did not receive it. Although not statistically significant, there was a trend towards a lower risk of hospitalization in antibody-treated patients. These findings are clinically significant, as lowering mortality and hospitalization due to COVID-19 disease is critical in alleviating the pandemic’s burden on the U.S. Additionally, antibody-treated patients also had better odds of fever resolution, dyspnea resolution, and improvement in POX, all indicators of clinical improvement [11].

The binary logistic analysis, which tends to describe the outcome based on baseline patient characteristics without comparing the treatment and the control group, suggested an association of higher mortality with what seems to be markers of more severe disease (for example, time to resolution of shortness of breath) and pro-coagulant activation such as D-dimer.

No serious adverse events occurred in any of the 162 patients in the LY-CoV555 group. Most of the adverse events were mild and transient, including diarrhea, nausea, headache, vomiting, and chills, and one patient with a mild rash. No change of vital signs was detected during the completion of the infusion. Therefore, the side effects related to infusions were relatively minor and were seen in 4.37% (7/160) of antibody-treated patients.

Originally the US-FDA issued an emergency use authorization (EUA) for monoclonal antibody therapy for the treatment of mild-to-moderate COVID-19 in adult and pediatric patients [29]. However, at the time of this study (November 2020 - January 2021), variants of the COVID-19 virus only had a prevalence of about 5% in the U.S. In March 2021, prevalence has increased to approximately 20%. Therefore, as of April 16, 2021, the FDA revoked the single use of LY-CoV555 after Eli Lilly requested to do so in order to prevent possible treatment failures. Lily released a new dual monoclonal antibody treatment, LY-CoV555 with LY-CoV016 (Etesevimab), which confers better protection against U.K. variant strains that are now predominant in the US [30].

Limitation

The major limitation of our study is its retrospective design. We also only used LY-CoV555 and not REGN-COV2. Lastly, there are some differences in baseline characteristics of the case and control group.

## Conclusions

Our analysis demonstrates that early treatment with monoclonal antibody LY-CoV555 was associated with decreased odds of mortality among high-risk patients in LTCFs with mild-to-moderate COVID-19 infection. Although not statistically significant, there was a trend toward a lower risk of hospitalization in LY-CoV555-treated patients. Further studies are needed to confirm these associations.
